# Sociodemographic and clinical variables associated with negative illness perception in patients newly diagnosed with rheumatoid arthritis, axial spondyloarthritis, or psoriatic arthritis—a survey based cross-sectional study

**DOI:** 10.1007/s00296-024-05553-0

**Published:** 2024-04-02

**Authors:** Luise Holberg Lindgren, Annette de Thurah, Tanja Thomsen, Merete Lund Hetland, Mette Aadahl, Sofie Bech Vestergaard, Sara Danshøj Kristensen, Bente Appel Esbensen

**Affiliations:** 1 Copenhagen Center for Arthritis Research (COPECARE)Center for Rheumatology and Spine DiseasesCentre for Head and Orthopedics Rigshospitalet, Glostrup, Denmark; 2 Department of Rheumatology, Aarhus University Hospital, Aarhus, Denmark; 3 Department of Clinical Medicine, Aarhus University, Aarhus, Denmark; 4 Center for Clinical Research and Prevention, Bispebjerg and Frederiksberg Hospital, Frederiksberg, Denmark; 5 Department of Clinical Medicine, University of Copenhagen, Copenhagen, Denmark

**Keywords:** Arthritis, Cross-sectional studies, Surveys and questionnaires, Self-management, Illness perception, Patient reported outcome measures

## Abstract

When newly diagnosed with inflammatory arthritis (IA), acquiring self-management skills is beneficial, to enhance quality of life. The personal beliefs and mental representations patients hold about their illness, known as illness perception, significantly influence the development of these skills. Recognizing characteristics that affect illness perception is key to identifying patients requiring additional support for the development of self-management skills. This study aimed at identifying the sociodemographic and clinical characteristics associated with a negative illness perception. This cross-sectional study was based on survey data from patients diagnosed for ≤ 2 years. The Brief Illness Perception Questionnaire (B-IPQ) was used to measure illness perception. After psychometric testing, we divided the B-IPQ into two domains: (1) a control domain and (2) a consequence domain. We performed logistic regression analyses with multiple imputations. A total of 1,360 patients (61% females) were included. Among them, 64%, 20%, and 16% were diagnosed with rheumatoid arthritis, psoriatic arthritis (PsA) and axial spondyloarthritis (axSpA), respectively. Younger patients with lower socioeconomic status, a diagnosis of PsA or axSpA, high disease activity (OR 3.026, CI 2.208;4.147), severe physical disability (OR 4.147. CI 2.883;6.007), severe pain (OR 3.034, CI 1.991;4.622), and severe fatigue (OR 2.612, CI 1.942;3.513) were significantly more likely to report having a negative illness perception. Younger patients with a higher symptom burden, increased disease activity, lower socioeconomic status, and a diagnosis of PsA or axSpA may require additional attention and support in rheumatology clinical practice to aid in the development of their self-management skills.

**Supplementary Information:**

The online version contains supplementary material available at 10.1007/s00296-024-05553-0.

## Background

Inflammatory arthritis constitutes a group of acute and chronic joint diseases characterized by joint pain, swelling and tenderness, caused by underlying inflammation [1–3]. In this study, we used the term inflammatory arthritis (IA) to cover the three most common chronic inflammatory arthritides: rheumatoid arthritis (RA), psoriatic arthritis (PsA) and axial spondyloarthritis (axSpA). All affect physical function and quality of life. The symptom burden can be severe and complex as symptoms can interact and be reinforcing [1–3].

Patients who are newly diagnosed with IA diseases are particularly challenged. In addition to being diagnosed with a chronic illness that includes lifelong treatment, many also experience changes in family roles, working life, and social relationships [4, 5]. According to the guidelines from the American College of Rheumatology (ACR) [6] and the European Alliance of Associations for Rheumatology (EULAR) [7], self-management advice and resources should be included in the routine management of IA. Self-management can be defined as the individual’s ability to manage symptoms, treatments, physical and psychosocial consequences, and lifestyle changes inherent in living with a chronic condition [8]. To develop self-management skills, newly diagnosed patients require regular appointments and available support from healthcare professionals (HPs) [5].

Several factors can affect patients’ ability to develop self-management skills. According to the common sense model (CSM) [9], illness perceptions (both cognitive and emotional) and coping responses are the determinants of illness-related behaviours and medical outcomes. The model includes emotional reactions and cognitive perceptions of a health problem that may explain how a person solves or manages health problems [9]. Thus, a person’s illness perception reflects the understanding of whether the disease is manageable or threatening, thereby influencing the ability to cope with the disease [10]. Positive illness perception has been shown to be a determinant of effective self-management in several chronic diseases [11, 12], whereas negative illness perceptions are associated with poor patient-reported outcomes over time [13]. Hence, according to the CSM, illness perception can play a significant role in predicting the development of effective self-management skills. Therefore, it is important to identify the factors associated with a negative illness perception, hence identifying patients who may require additional attention and assistance in developing self-management skills.

We hypothesised that high disease activity and low socioeconomic status were associated with negative illness perception.

Therefore, the aim of the present study was to identify specific sociodemographic and clinical characteristics in patients newly diagnosed with IA who have a negative perception of their illness.

## Methods

### Study design and data collection

This is a cross-sectional study based on data from the Mental Health in Inflammatory Arthritis (MaIA) study [14]—a national cross-sectional study conducted in January–February 2022 among Danish patients with IA. The response rate was approximately 33% (12,713/38,161).

As described in the MaIA study [14] a preliminary test for face validity was conducted. This involved engaging 10 patients from two distinct rheumatology outpatient clinics in Denmark. To meticulously assess and refine the questionnaire's relevance and comprehensibility from the patient's perspective, semi-structured interviews were conducted using the “think aloud” method.

Eligible patients were identified through the Danish Rheumatology Database, DANBIO [15], which has a high completeness rate (~ 90%). They were invited to answer the questionnaire through their official digital mailbox (‘e-Boks’). In the case of nonresponse, a reminder was sent 10 days after the initial invitation. The index date was the day the participants responded to the questionnaire.

Study data were collected and managed using REDCap electronic data capture tools [16]. The project was accepted and registered by the Danish Data Protection Agency (journal number.: P-2021–509).

We followed the STROBE guidelines [17] for reporting cross-sectional studies.

### Participants

The inclusion criteria for participation in the MaIA study were adult persons (≥ 18 years), with one of the following diagnoses: RA (ICD10 diagnoses: M05.9, M06.0, M06.9), axSpA (ICD10 diagnoses: M45.9, M46.1, M46.8, M46.9) or PsA (ICD10 diagnoses: M073.A, M073.B).

Patients were excluded based on the following criteria: lack of registration to the official digital mailbox, e-Boks.

For the present study, we included data from patients who had been diagnosed within 2 years, which corresponded to 1,360 respondents. There were no exclusion criteria.

## Data sources/Measurements

### Primary outcome

*Illness perception* was measured using the Brief Illness Perception Questionnaire (B-IPQ) (16). The B-IPQ originated from the Illness Perception Questionnaire (IPQ) and the Illness Perception Questionnaire-Revised (IPQ-R) and is designed to provide a quick and straightforward assessment of illness perceptions [18]. The B-IPQ [9, 19] consists of eight items graded on a linear 0–10 response scale that is used to measure cognitive and emotional illness representations. Each item of the B-IPQ assesses one dimension of illness perception (consequences, timeline, personal control, treatment control, illness identity, concern, coherence, and emotional representation). Item 9 is a causal item and is answered in an open-ended question; this item was excluded from the analysis [9, 19].

Five items represent ‘cognitive illness representations’ (items 1–5), two represent ‘emotional representations’ (items 6 and 8), and one represents ‘illness comprehensibility’ (item 7). For B-IPQ items 1, 2, 5, 6 and 8, higher scores indicate more negative illness perceptions. For B-IPQ items 3, 4 and 7, lower scores indicate more negative illness perceptions [9, 19].

Having a negative illness perception means that the individual perceives one’s illness as threatening to overall health and life e.g., because of experiencing more symptoms due to the illness or being more concerned about the illness [18].

The B-IPQ has been used in different populations, encompassing various age groups, types of illnesses, countries, and languages. Psychometric evaluations have demonstrated good concurrent and predictive validity, as well as sensitivity to change [20].

### Explanatory variables

#### Demographic and socioeconomic variables

The demographic variables included age, sex, household income, educational level, connection to the labour market and cohabitant status.

Age and sex were determined based on CPR number (a personal identification number provided to every Danish citizen) and stored in the Civil Registration System, Denmark. Household income, educational level, connection to the labour market and cohabitant status were based on self-assessment. We used the International Standard of Classification of Education [21]. Connection to the labour market was categorised as ‘available’ or ‘not available’. ‘Available’ was defined to cover employed (full-time, part-time or on special terms), unemployed and vocational training/student. ‘Not available’ covered retired, being on sick leave benefit and early retirement pension.

#### Clinical variables

Clinical variables included disease duration, disease activity, physical function, pain, and fatigue.

*Disease duration* was displayed in years and calculated as the time from the first registration in DANBIO until the index date.

*Disease activity* was also retrieved from DANBIO using the measurement closest to the index date. Disease activity in RA was measured using the Disease Activity Score in 28 joints with an erythrocyte sedimentation rate (DAS28) [22]. For measuring disease activity in patients with axSpA, we used the Bath Ankylosing Spondylitis Disease Activity Index (BASDAI) [23]. To measure disease activity in patients with PsA, we used the Disease Activity Index for PSoriatic Arthritis (DAPSA) [24].

*Physical function* was measured using the Multidimensional Health Assessment Questionnaire (MD-HAQ) [25]. 

*Pain* was measured using the visual analogue scale (VAS) [26]. 

*Fatigue* was measured using the VAS fatigue scale [27]. For both MD-HAQ and VAS, a higher score indicates worse conditions.

## Statistics

Initially, we examined the prerequisites for calculating a total score of the B-IPQ [28] by checking the internal consistency. We recorded the responses in item 3, 4 and 7 to be in the same direction as the other items, and performed and analysed Cronbach’s alpha, mean interitem correlations (MIICs) and exploratory and confirmatory factor analysis (Supplementary material Table A and Table B, Figs. 1. and 2). This showed a two-factor structure, from which we defined the consequence and control domain (Supplementary material Table C). *The consequence domain* captures the patient’s perspective on the symptom burden (illness identity) and the impact of the disease on their health and life. It encompasses the level of concern and emotional impact experienced by the patient. Therefore, this domain reflects the patient’s thoughts and emotions regarding their current health status. *The control domain* compromises items that represent the extent to which the patient believes that they can recover from or control the illness, as well as their understanding of the disease. Thus, this domain reflects the patient’s thoughts and emotions regarding their capacity to influence their health status.

Demographic data were presented with simple descriptive statistics such as mean, standard deviations, frequency, and percentages.

All missing data have been handled by multiple imputations using a three-step procedure in Statistical Analysis Software (SAS) [29]. We assumed that all data were missing at random and used the multivariate normal distribution method because we assumed that all the variables in the imputation model had a joint multivariate normal distribution [29]. We made 50 imputations. The highest amount of missing data was in the DAPSA score, where the proportion was 23%; in B-IPQ, it was 2%. The pooled imputed data were used for the analyses described below.

To define the patients with the most negative illness perceptions, we divided the B-IPQ scores in the consequence and control domains into tertiles using the upper tertile as the cut-off to define patients with high consequence within the consequence domain and the lower tertile to define patients with low control within the control domain. Because there are no official cut-offs for defining negative illness perception, tertiles provided the best power and best possible balance of the number of patients per group [30].

Also, measures for disease activity (DAS28, DAPSA and BASDAI) were divided into tertiles, where the lower tertile was used as the reference group in the analysis. MD-HAQ was categorised into the following three categories: no to very mild disability (scores 0–0.3), mild disability (0.3–1.0) and moderate to severe disability (> 1.0).

We categorised pain based on previously used cut-offs, where ≤ 3.5 indicated mild pain; the cut-off points for moderate pain were 3.5–7.5, and over 7.5 represented severe pain [26]. Clinically relevant fatigue has previously been defined as a VAS score ≥ 20 mm and high fatigue scores as a VAS score ≥ 50 mm [31]. Therefore, we categorised fatigue as follows: no or mild fatigue < 20 mm, moderate fatigue as ≥ 20 mm to < 50 mm and high fatigue as ≥ 50 mm.

We used logistic regression analyses to identify the factors associated with having a negative illness perception and presented the estimates as odds ratios (ORs) with 95% confidence intervals. A series of multiple logistic regression analyses were performed in each domain to examine associations of negative illness perceptions and the following explanatory variables: age, sex, household income, educational level, cohabitant status, duration (time after diagnosis), disease activity, physical function, pain, and fatigue. First, we examined crude associations with sociodemographic and clinical characteristics, and then, we examined the associations adjusted for sex and age (model 1). The significance level for all analyses was set at 5%. Sensitivity analyses were carried out by exploring whether tendencies in the logistic regression analysis were similar when the variables were modelled in linear regression models.

To improve generalisability, we used stepwise selection logistic regression to identify the model with the greatest associations with high consequence and low control [32]. The method is stable when the sample size > 50 per variable. We used a 0.3 significance level for variable entry into the model, hence defining the model with the highest area under the curve (AUC).

All statistical analyses were carried out in SAS Enterprise Guide 8.3.

### Patient research partner

We involved a patient research partner [33] in all phases of the study.

## Results

### Study population characteristics

In total 1,360 patients were included in this study, from those, 64% were diagnosed with RA, 20% with PsA and 16% with axSpA. Regarding age, 14% were between 18 and 40 years old, 40% were between 41 and 60 years old, and 46% were older than 61 years (Table 1).

**Table 1:** Characteristics of the study population

Variables Demographics	Total *n* = 1,360	RA *n* = 865 (64%)	PsA *n* = 275 (20%)	axSpA *n* = 220 (16%)
Sex				
Females, *n* (%)	835 (61)	557 (64)	163 (59)	115 (52)
Males, *n* (%)	525 (39)	308 (36)	112 (41)	105 (48)
Age years, mean, (SD)	57.17 (15.28)	61.24 (14.20)	54.38 (13.21)	44.61 (14.21)
Young adult 18–40,* n* (%)	195 (14)	77 (9)	37 (13)	81 (37)
Adult 41–60, *n* (%)	538 (40)	286 (33)	145 (53)	107(49)
Senior adult > 60, *n* (%)	627(46)	502(59)	93 (34)	32(14)
Household income in EURO				
Declined to answer, *n* (%)	176 (13)	116 (13)	32 (11)	28 (13)
< 67,000, *n* (%)	664 (49)	463 (54)	123 (45)	78 (35)
> 67,000, *n* (%)	520 (38)	286 (33)	120 (44)	114 (52)
Educational level				
Basic, secondary, short, *n* (%)	574 (42)	351 (40)	121 (44)	102 (46)
Intermediate and long > 2 years, *n* (%)	779 (57)	509 (59)	152(55)	118 (54)
Missing 7, *n* (%)	7 (1)	5 (1)	2 (1)	-
Available to the labour market				
Yes, *n* (%)	727 (54)	388 (45)	167 (61)	172(78)
No, *n* (%)	630 (46)	475 (55)	107 (39)	48 (22)
Missing, *n* (%)	3 (0)	2 (0)	1 (09)	-
Cohabitant status—Living with someone				
Yes, *n* (%)	1088 (80)	672 (78)	229 (73)	187 (85)
No, *n* (%)	272 (20)	193 (22)	46 (17)	33 (15)
Clinical variables				
Disease duration				
< 1 year, *n* (%)	617 (45)	374 (43)	125 (45)	118 (53)
> 1 ≤ 2 years, *n* (%)	743 (55)	491 (57)	150 (55)	102 (47)
Disease activity				
RA^1^ (DAS28)^2^, mean (SD)	2.92 (1.33)		––	––
Tertile 0.96–1.96, *n* (%)		241 (28)		
Tertile 1.97–3.40, *n* (%)		241 (28)		
Tertile 3.41–7.99, *n* (%)		241 (28)		
Missing, *n* (%)		142 (16)		
PsA^3^ (DAPSA)^4^, mean (SD)	15.11 (11.12)	––		––
Tertile 0.29–8.40, *n* (%)			70 (25)	
Tertile 8.65–17.40, *n* (%)			71 (26)	
Tertile 17.80–49.46, *n* (%)			70 (25)	
Missing, *n* (%)			64 (23)	
axSpA^5^ (BASDAI)^6^, mean (SD)	41.60 (24.12)	––	––	
Tertile 0–24.8, *n* (%)				67 (30)
Tertile 25.6–55.2, *n* (%)				67 (30)
Tertile 56.2–100.0, *n* (%)				67 (30)
Missing, *n* (%)				19 (9)
Physical function (MD-HAQ)^7^, mean (SD)	0.47 (0.47)	0.45 (0.46)	0.55 (0.48)	0.50 (0.47)
< 0.3 none to very mild disability, *n* (%)	498 (36)	345 (40)	77 (28)	76 (35)
0.3–1.0 mild disability, *n* (%)	500 (37)	298 (34)	110 (40)	92 (42)
> 1.0 moderate to severe disability, *n* (%)	187 (14)	114 (13)	44 (16)	29 (13)
Missing 175, *n* (%)	175 (13)	108 (13)	44 (16)	23 (10)
Pain (VAS)^8^, mean (SD)	37.83 (27.57)	34.99 (27.39)	42.62 (25.35)	43.09 (29.27)
No or mild ≤ 3.5, *n* (%)	795 (58)	535 (62)	148 (54)	112 (51)
Moderate 3.5–7.5, *n* (%)	438 (32)	258 (30)	103 (37)	77 (35)
Severe > 7.5, *n* (%)	127 (9)	72 (8)	24 (9)	31(14)
Fatigue (VAS)^9^, mean (SD)	44.17 (29.80)	40.77 (29.59)	50.78 (29.18)	49.46 (29.38)
No or mild ≤ 20, *n* (%)	490 (36)	346 (40)	83 (30)	61 (28)
Moderate 21–50, *n* (%)	312 (23)	193 (22)	61 (22)	58 (26)
Severe ≥ 50, *n* (%)	558 (41)	326 (38)	131 (48)	101 (46)

^1^Rheumatoid arthritis

^2^Disease activity score in 28 joints with erythrocyte sedimentation rate

^3^Psoriatic arthritis

^4^Disease activity index for psoriatic

^5^Arthritis axial spondyloarthritis

^6^Bath ankylosing spondylitis disease activity index

^7^Multidimensional health assessment questionnaire

^8^Visual analogue scale

A total of 42% had basic, secondary, or short education, and 80% were cohabitating. Compared with RA and PsA, patients with axSpA were younger, had a higher household income, possessed higher levels of education and were more available to the labour market (78% compared with 45% in the RA group).

In total, 45% of the patients had been diagnosed within the last year, and 86% had an MD-HAQ score > 1 and, overall, low disease activity that was evenly distributed between diagnoses. Patients with RA had less pain and fatigue than those in the other two diagnostic groups, while patients diagnosed with axSpA included a larger proportion of patients with severe pain.

### Sociodemographic and clinical characteristics of patients with high levels of illness perception

#### Consequence domain

Generally, patients diagnosed with PsA, or axSpA had significantly higher odds of perceiving high consequences of the disease compared with those diagnosed with RA (Table 2). In addition, younger age, lower income, not being available to the labour market and living alone were significantly associated with experiencing high disease consequences when adjusted for sex and age.

**Table 2:** Variables associated with high consequences (consequence domain)

Variables	High consequence		High consequence—sex and age adj	
	OR (95% Cl)	*p*-value	OR (95% Cl)	*p*-value
Sex				
Male	Reference		Reference	
Female	**1.32 (1.05; 1.67)**	**0.02**	1.20 (0.94; 1.53)^A^	0.15
Age				
Young adult 18–40 *n* (%)	**2.63 (1.88; 3.67)**	** < 0.01**	**2.54 (1.81; 3.55)**^**B**^	** < 0.01**
Adult 41–60	**2.18 (1.70; 2.80)**	** < 0.01**	**2.15 (1.68; 2.76)**^**B**^	** < 0.01**
Senior adult > 60	Reference		Reference	
Diagnosis				
Rheumatoid arthritis	Reference		Reference	
Psoriatic arthritis	**2.00 (1.48; 2.71)**	** < 0.01**	**1.55 (1.11; 2.16)**	**0.01**
Axial spondyloarthritis	**1.59 (1.20; 2.10)**	** < 0.01**	**1.39 (1.04; 1.85)**	**0.03**
Household income (in EURO)				
Decline to answer	1.18 (0.82; 1.68)	0.38	**1.59 (1.07; 2.36)**	**0.02**
< 67,000	1.15 (0.90; 1.46)	0.26	**1.84 (1.38; 2.45)**	** < 0.01**
> 67,000	Reference		Reference	
Educational level				
Basic, second and short	1.03 (0.82; 1.28)	0.81	1.16 (0.91; 1.49)	0.23
Intermediate and long (> 2 years)	Reference		Reference	
Available to the labour market				
Yes (%)	Reference		Reference	
No (%)	1.04 (0.83; 1.30)	0.72	**2.74 (1.95; 3.84)**	** < 0.01**
Cohabitant status				
Living with someone	Reference		Reference	
Living alone	1.24 (0.95; 1.63)	0.12	**1.46 (1.08; 1.97)**	**0.02**
Disease duration				
< 1 year	1.02 (0.81; 1.27)	0.89	0.98 (0.77; 1.24)	0.85
> 1 ≤ 2 years	Reference		Reference	
Disease activity				
RA^1^ (DAS28)^2^				
Tertile 0.96–1.96	Reference		Reference	
Tertile 1.97–3.40	**2.02(1.36; 3.00)**	** < 0.01**	**1.91 (1.25; 2.94)**	**0.02**
Tertile 3.41–7.99	**3.81 (2.60; 5.59)**	** < 0.01**	**3.61 (2.38; 5.46)**	** < 0.01**
PsA^3^ (DAPSA)^4^				
Tertile 0.29–8.40	Reference		Reference	
Tertile 8.65–17.40	**3.23 (1.73; 6.03)**	** < 0.01**	**2.06 (1.00; 4.26)**	**0.05**
Tertile 17.80–49.46	**2.48 (1.33; 4.64)**	** < 0.01**	1.41 (0.66; 2.99)	0.38
AxSpA^5^ (BASDAI)^6^				
Tertile 0–24.8	Reference		Reference	
Tertile 25.6–55.2	**2.60 (1.29; 5.24)**	**0.01**	1.72 (0.72; 4.10)	0.28
Tertile 56.2–100.0	**7.08 (3.41; 14.70)**	** < 0.01**	**4.04 (1.65; 9.93)**	**0.01**
Pooled disease activity				
1. Tertile	Reference		Reference	
2. Tertile	**2.32 (1.72; 3.13)**	** < 0.01**	**2.27 (1.65; 3.14)**	** < 0.01**
3. Tertile	**3.78 (2.82; 5.08)**	** < 0.01**	**3.64 (2.65; 5.01)**	** < 0.01**
Physical function (MD-HAQ)^7^				
< 0.3 zero to very mild disability	Reference		Reference	
0.3–1.0 mild disability	**2.29 (1.76; 2.98)**	** < 0.01**	**2.16 (1.63; 2.86)**	** < 0.01**
> 1.0 moderate to severe disability	**5.48 (3.90; 7.69)**	** < 0.01**	**5.05 (3.51; 7.27)**	** < 0.01**
Pain (VAS)^8^				
No or mild ≤ 3.5	Reference		Reference	
Moderate 3.5–7.5	**2.76 (2.15; 3.54)**	** < 0.01**	**2.59 (1.98; 3.38)**	** < 0.01**
Severe > 7.5	**8.55 (5.60; 13.04)**	** < 0.01**	**7.00 (4.45; 11.02)**	** < 0.01**
Fatigue (VAS)^8^				
No or mild ≤ 20	Reference		Reference	
Moderate 21–50	**1.54 (1.11; 2.14)**	**0.01**	1.36 (0.95; 1.92)	0.09
Severe ≥ 50	**4.17 (3.17; 5.49)**	** < 0.01**	**3.33 (2.48; 4.48)**	** < 0.01**

^1^Rheumatoid Arthritis

^2^Disease Activity Score in 28 joints with erythrocyte sedimentation rate

^3^Psoriatic Arthritis

^4^Disease Activity Index for PSoriatic Arthritis

^5^Axial Spondyloarthritis

^6^Bath Ankylosing Spondylitis Disease Activity Index

^7^Multidimensional Health Assessment Questionnaire

^8^Visual Analogue Scale

Significant results are shown in **bold**

^A^Not adjusted for sex

^B^Not adjusted for age

Patients with the highest levels of disease activity perceived the highest consequence (Adjusted (Adj) OR = 3.64, CI: 2.65; 5.01). This pattern was also applied to the groups with high disability, high fatigue, and high pain (Adj OR = 7.00 CI: 4.45; 11.02). The stepwise selection regression analysis with high consequence as the response variable revealed that a model consisting of pain, age, physical function, availability to the labour market, fatigue, cohabitant status, diagnosis and disease activity were associated with the experience of high consequences of the disease (Supplementary material Table D1). The model demonstrated an AUC value of 0.75.

#### Control domain

In the control domain (Table 3), younger age was associated with experience of low control (Adj OR = 2.11, CI: 1.50;2.96). Also, patients with low household income, low educational level, being not available to the labour market and high levels of pain, fatigue and disability were more likely to experience low control when adjusted for age and sex.

**Table 3:** Variables associated with low control (control domain)

Variables	Low control		Low control—sex and age adj	
	OR (95% Cl)	*p*-value	OR (95% Cl)	*p*-value
Sex				
Male	Reference		Reference	
Female	1.14 (0.90; 1.44)	0.28	1.05 (0.82; 1.34)^A^	0.72
Age				
Young adult 18–40	**2.13 (1.52; 2.98)**	** < 0.01**	**2.11(1.50; 2.96)**^**B**^	** < 0.01**
Adult 41–60	**1.66 (1.29; 2.14)**	** < 0.01**	**1.65 (1.28; 2.13)**^**B**^	** < 0.01**
Senior adult > 60	Reference		Reference	
Diagnosis				
Rheumatoid arthritis	Reference		Reference	
Psoriatic arthritis	**1.72 (1.27; 2.35)**	** < 0.01**	**1.44 (1.00; 2.08)**	**0.05**
Axial spondyloarthritis	**1.63 (1.22; 2.17)**	** < 0.01**	**1.50 (1.10; 2.05)**	**0.01**
Household income in EURO				
Decline to answer	1.34 (0.93; 1.93)	0.11	**1.64 (1.09; 2.45)**	**0.02**
< 67,000	1.21 (0.94; 1.55)	0.14	**1.59 (1.19; 2.13)**	** < 0.01**
> 67,000	Reference		Reference	
Educational level				
Basic and secondary and short	1.19 (.0.94; 1.50)	0.28	**1.31 (1.01; 1.69)**	**0.04**
Intermediate and long (> 2 years)	Reference		Reference	
Available to the labour market				
Yes (%)	Reference		Reference	
No (%)	0.92 (0.73; 1.16)	0.52	**1.87 (1.34; 2.61)**	** < 0.01**
Cohabitant status				
Living with someone	Reference		Reference	
Living alone	1.02 (0.77; 1.36)	0.88	1.22 (0.89; 1.66)	0.22
Disease duration				
< 1 year	**1.26 (1.00; 1.59)**	**0.05**	1.20 (0.94; 1.53)	0.14
> 1 ≤ 2 years	Reference		Reference	
Disease activity				
RA^1^ (DAS28)^2^				
Tertile 0.96–1.96	Reference		Reference	
Tertile 1.97–3.40	1.27 (0.86; 1.89)	0.24	1.29 (0.84; 1.00)	0.25
Tertile 3.41–7.99	**2.58 (1.77; 3.75)**	** < 0.01**	**2.88 (1.90; 4.37)**	** < 0.01**
PsA^3^ (DAPSA)^4^				
Tertile 0.29–8.40	Reference		Reference	
Tertile 8.65–17.40	**2.15 (1.15; 4.00)**	**0.02**	1.70 (0.80; 3.65)	0.17
Tertile 17.80–49.46	**2.24 (1.21; 4.18)**	**0.01**	1.79 (0.82; 3.90)	0.15
AxSpA^5^ (BASDAI)^6^				
Tertile 0–24.8	Reference		Reference	
Tertile 25.6–55.2	1.97 (0.94; 4.15)	0.07	1.46 (0.54; 3.96)	0.45
Tertile 56.2–100.0	**6.99 (3.33; 14.69)**	** < 0.01**	**7.63 (2.51; 23.15)**	** < 0.01**
Pooled disease activity				
1. Tertile	Reference		Reference	
2. Tertile	**1.55 (1.14; 2.10)**	**0.01**	**1.44 (1.04; 1.99)**	**0.03**
3. Tertile	**2.93 (2.19;3.93)**	** < 0.01**	**3.03 (2.21; 4.15)**	** < 0.01**
Physical function (MD-HAQ)^7^		** < 0.01**		
< 0.3 zero to very mild disability	Reference		Reference	
0.3–1.0 mild disability	**2.50 (1.91; 3.29)**	** < 0.01**	**2.67 (1.99; 3.57)**	** < 0.01**
> 1.0 moderate to severe disability	**3.83 (2.72; 5.39)**	** < 0.01**	**4.15 (2.88; 6.01)**	** < 0.01**
Pain (VAS)^8^				
No or mild ≤ 3.5	Reference		Reference	
Moderate 3.5–7.5	**2.07 (1.61; 2.66)**	** < 0.01**	**1.99 (1.52; 2.61)**	** < 0.01**
Severe > 7.5	**3.19 (2.17; 4.68)**	** < 0.01**	**3.03 (1.99; 4.62)**	** < 0.01**
Fatigue (VAS)^8^				
No or mild ≤ 20	Reference		Reference	
Moderate 21–50	1.17 (0.83; 1.63)	0.37	1.06 (0.74; 1.52)	0.74
Severe ≥ 50	**2.90 (2.21; 3.81)**	** < 0.01**	**2.61 (1.94; 3.51)**	** < 0.01**

^1^Rheumatoid Arthritis

^2^Disease Activity Score in 28 joints with erythrocyte sedimentation rate

^3^Psoriatic Arthritis

^4^Disease Activity Index for PSoriatic Arthritis

^5^Axial Spondyloarthritis

^6^Bath Ankylosing Spondylitis Disease Activity Index

^7^Multidimensional Health Assessment Questionnaire

^8^Visual Analogue Scale

Significant results are shown in **bold**

^A^Not adjusted for sex

^B^Not adjusted for age

Disease duration showed nonsignificant results in the adjusted analysis (Adj OR = 1.20, CI: 0.94;1.53).

The stepwise regression (Supplementary material Table D2) showed that a model with fatigue, physical function, age, disease activity, diagnosis and household income accounted for the most variance in the response variable and had an AUC value of 0.70.

## Discussion

In the present study, we identified specific sociodemographic and clinical characteristics in patients newly diagnosed with IA and negative illness perception. We found that lower age, high disease activity, severe physical, high-level pain, high level of fatigue, lower household income, not being available to the labour market and a diagnosis of PsA or axSpA were significantly associated with perceptions of low control and a high level of consequence. High symptom burden and high disease activity demonstrated higher odds compared with the socioeconomic variables, implying that these variables were of greater importance. These results align with findings from several studies conducted on chronic diseases that have consistently indicated a correlation between negative illness perception and higher levels of disability, as well as increased pain. In addition, pain has been found to be the domineering factor in the experience and intensity of fatigue [34–37]. These findings are also consistent with the CMS model, and to some degree also to our hypothesis. Our hypothesis posited that high disease activity and low socioeconomic status is associated with negative illness perception. Our findings confirmed the association between high disease activity and negative illness perception and confirmed to some degree, that variables indicating low socioeconomic status, was associated with negative illness perception.

A cohort study of patients with RA [13] demonstrated that patients’ perceptions of their illness generally improve to a certain degree during the first year following diagnosis. Therefore, a surprising result in our study was that the duration of the illness displayed no significant association with either the consequence domain or control domain. This observation could imply that patients need more than a year, to accept and adjust to their IA diagnosis, or that a potential decrease in illness perception was unclear in our data because of our cross-sectional design.

Based on the results from other studies [38, 39], we initially anticipated that being female would be a risk factor. However, no significant associations were found in the adjusted analysis. Additionally, we did not identify a clear association with educational level, which was also unexpected considering such associations have been found in patients with myasthenia gravis, chronic prostatitis and ischaemic stroke [40].

Patient’s illness perceptions are influenced by various factors, including the initial shock of being diagnosed with a chronic condition, self-assessed health status, role identities and cultural influences [18]. When patients are newly diagnosed, they often rely on available information, such as conversations with others and guidance from HPs, to form their illness perception. These perceptions, in turn, trigger a self-regulation process aimed at mitigating the perceived health threat [41]. Thus, illness perceptions significantly impact a patient’s ability to adapt to their condition, and the success of these adaptions subsequently influences their later illness perceptions. Several studies have shown the profound impact of illness perceptions on outcomes [42], with evidence indicating that modifying these perceptions can bring about changes in outcomes. Research conducted across various conditions has illustrated that interventions aimed at promoting positive illness perceptions and beliefs can lead to improvements in social and occupational limitations, symptoms, self-management and overall quality of life [43–45]. Nevertheless, the association between health beliefs, illness perceptions and adherence to medications and lifestyle recommendations [13] implies the importance of implementing interventions early in the disease course before negative perceptions become deeply ingrained and difficult to change.

The present study identified several key factors indicating the necessity for additional attention and support in the development of self-management. Thus, recognising these factors allows for targeted support from HPs to be provided, thereby facilitating the successful development of self-management skills. Our results strongly suggest that managing high symptom burden and high disease activity are critical for how patients perceive their illness. Treatment and symptom management, along with diminishing psychosocial consequences, constitute the fundamental aspects of core self-management skills [8] and confirm the relevance of applying interventions to support the development of self-management in rheumatology clinical practice.

Unfortunately, some of the above-mentioned characteristics are commonly found in patients who are less likely to engage in self-management interventions [46, 47]. Nonparticipants are often characterised by lower socioeconomic status and younger age. Thus, these socioeconomic- and age-related pitfalls in participation in and benefits derived from self-management interventions place substantial demands on the healthcare system. Addressing these specific challenges necessitates tailored and differentiated interventions.

### Strengths and limitations

It is essential to recognise some limitations. As this is a cross-sectional study, we could establish associations but not draw any conclusions regarding causal inference. Although we have adjusted for potential available confounders, it is worth noting that unmeasured confounders, such as comorbidity or psychological distress, could contribute to increased negative illness perception and may have influenced our results. In addition, the choice of working with tertiles can be seen as a limitation because data are lost when categorising, but it makes the results easier to interpret. Approximately one-third of the patients responded to the questionnaire, resulting in an acceptable response rate of around 33%. However, it is important to recognise that this response rate may introduce selection bias. Potentially, patients experiencing higher levels of anxiety and depression may be less inclined to respond to the questionnaire or patients in remission may perceive it as less relevant. Therefore, as part of the original MaIA study [14], a nonresponder analysis was conducted revealing that nonresponders were younger, had a higher functional disability as indicated by a higher MD-HAQ score and that more patients were diagnosed with axSpA. Because these factors also characterised the patients with the most negative illness perceptions, our data may have a lower prevalence of these patients, so our analysis may have underestimated the association.

The present study has several strengths. To the best of our knowledge, this is the first study to investigate illness perception in a population of patients newly diagnosed with RA, axSpA and PsA. Therefore, we conducted thorough psychometric testing. Our psychometric testing of the dimensional structure revealed a two-factor structure. This structure was also identified in previous studies of patients with heart failure, cancer and diabetes [38, 48, 49]. It is worth noting, however, that further examination of this structure remains necessary because another study [50] identified a factor structure compromising both cognitive and emotional components, as described by Broadbent [18]. Often, the B-IPQ has been used on the item level. This provides a nuanced picture; we did, however, wish to simplify the analysis and results to make the results easier to interpret and use in clinical practice. Therefore, we used a two-factor structure, as defined by our exploratory and confirmatory factor analysis.

All the included variables in the current study were measured using validated reliable measurement instruments. Additionally, to assess disease activity, our data were linked with the reliable Danish register DANBIO. Another strength lies in the large number of patients, with the survey distributed to all Danish patients registered in the DANBIO register who had a digital mailbox, hence encompassing approximately 38,000 patients. Finally, the utilisation of an electronic survey questionnaire ensured accurate data collection.

## Conclusion

In conclusion, our findings indicate that younger age, lower household income, not being available to the labour market, a diagnosis of PsA or axSpA, high disease activity, severe physical disability, high levels of pain and fatigue were all significantly associated with patients experiencing low control and a high level of consequence.

Notably, variables related to disease activity and symptoms exhibited a stronger association with negative illness perception than sociodemographic variables. Therefore, patients with these characteristics may require targeted attention and support within rheumatology clinical practice to facilitate the successful development of self-management skills.

## Open access funding

Open access funding is provided by the Royal Library, Copenhagen University Library.

### Supplementary Information

Below is the link to the electronic supplementary material.

Supplementary file1 (DOCX 304 KB)

## Data Availability

Interested parties may obtain access to the data by making a reasonable request to the corresponding author.
