# Supplementary material for: Sociodemographic and clinical variables associated with negative illness perception in patients newly diagnosed with rheumatoid arthritis, axial spondyloarthritis, or psoriatic arthritis—a survey based cross-sectional study
Source: Rheumatol Int. 2024 Apr 2;44(6):1119–31. doi: 10.1007/s00296-024-05553-0 (PMC11108915; doi:10.1007/s00296-024-05553-0)
Supplement: Supplementary file 1 — Supplementary file1 (DOCX 304 KB) [file 296_2024_5553_MOESM1_ESM.docx]

## Supplementary material

**Table A. Mean, standard deviation, correlation, and Cronbach coefficient alpha for B-IPQ items**

| **B-IPQ Item sumscale** | **Total N**  Mean (SD) | **RA**  Mean (SD) | **PsA**  Mean (SD) | **axSpA**  Mean (SD) | **Correlation with total** | **Alpha** |
| --- | --- | --- | --- | --- | --- | --- |
| **Item 1 – Consequences** | 5.0 (2.9) | 4.7 (2.9) | 5.5 (2.9) | 5.8 (2.7) | 0.71 | 0.76 |
| **Item 2 – Timeline** | 8.3 (2.6) | 8.2 (2.7) | 8.4 (2.5) | 8.5 (2.5) | 0.20 | 0.83 |
| **Item 3 – Personal control** | 5.5 (2.9) | 5.8 (2.9) | 5.0 (3.0) | 4.6 (3.1) | 0.64 | 0.77 |
| **Item 4 – Treatment control** | 7.4 (2.5) | 7.7 (2.3) | 6.9 (2.7) | 6.9 (2.6) | 0.36 | 0.81 |
| **Item 5 – Illness identity** | 5.2 (2.7) | 4.9 (2.7) | 5.7 (2.5) | 5.6 (2.6) | 0.65 | 0.77 |
| **Item 6 – Concern** | 5.6 (2.9) | 5.4 (2.9) | 6.0 (2.7) | 6.1 (2.7) | 0.69 | 0.77 |
| **Item 7 – Coherence** | 5.9 (2.9) | 6.1 (2.9) | 5.7 (2.7) | 5.7 (2.8) | 0.32 | 0.82 |
| **Item 8 – Emotional representation** | 4.9 (3.1) | 4.6 (3.1) | 5.4 (2.9) | 5.4 (3.1) | 0.67 | 0.77 |
| **Total coefficient alpha** |  |  |  |  |  | 0.811344 |

**Table B. Correlation and Cronbach’s coefficient alpha of the consequence domain and control domain**

| **B-IPQ item** | **Total domain**  **correlation** | **Cronbach’s alpha** | **RA correlation** | **Cronbach’s alpha** | **PsA correlation** | **Cronbach’s alpha** | **axSpA correlation** | **Cronbach’s alpha** |
| --- | --- | --- | --- | --- | --- | --- | --- | --- |
| **Consequence domain** |  |  |  |  |  |  |  |  |
| **Item 1 – Consequences** | 0.75 | 0.807 | 0.755 | 0.811 | 0.787 | 0.818 | 0.666 | 0.740 |
| **Item 5 – Illness identity** | 0.69 | 0.836 | 0.679 | 0.842 | 0.712 | 0.849 | 0.632 | 0.758 |
| **Item 6 – Concern** | 0.72 | 0.823 | 0.730 | 0.821 | 0.744 | 0.836 | 0.583 | 0.779 |
| **Item 8 – Emotional representation** | 0.69 | 0.834 | 0.697 | 0.836 | 0.694 | 0.856 | 0.630 | 0.761 |
| **Total Cronbach’s alpha** |  | **0.863** |  | **0.865** |  | **0.875** |  | **0.808** |
| **Control domain** |  |  |  |  |  |  |  |  |
| **Item 3 – Personal control** | 0.54 | 0.495 | 0.543 | 0.532 | 0.527 | 0.471 | 0.466 | 0.297 |
| **Item 4 – Treatment control** | 0.48 | 0.585 | 0.512 | 0.591 | 0.436 | 0.621 | 0.347 | 0.500 |
| **Item 7 – Coherence** | 0.44 | 0.627 | 0.460 | 0.647 | 0.471 | 0.577 | 0.314 | 0.547 |
| **Total Cronbach’s alpha** |  | **0.668** |  | **0.685** |  | **0.665** |  | **0.562** |


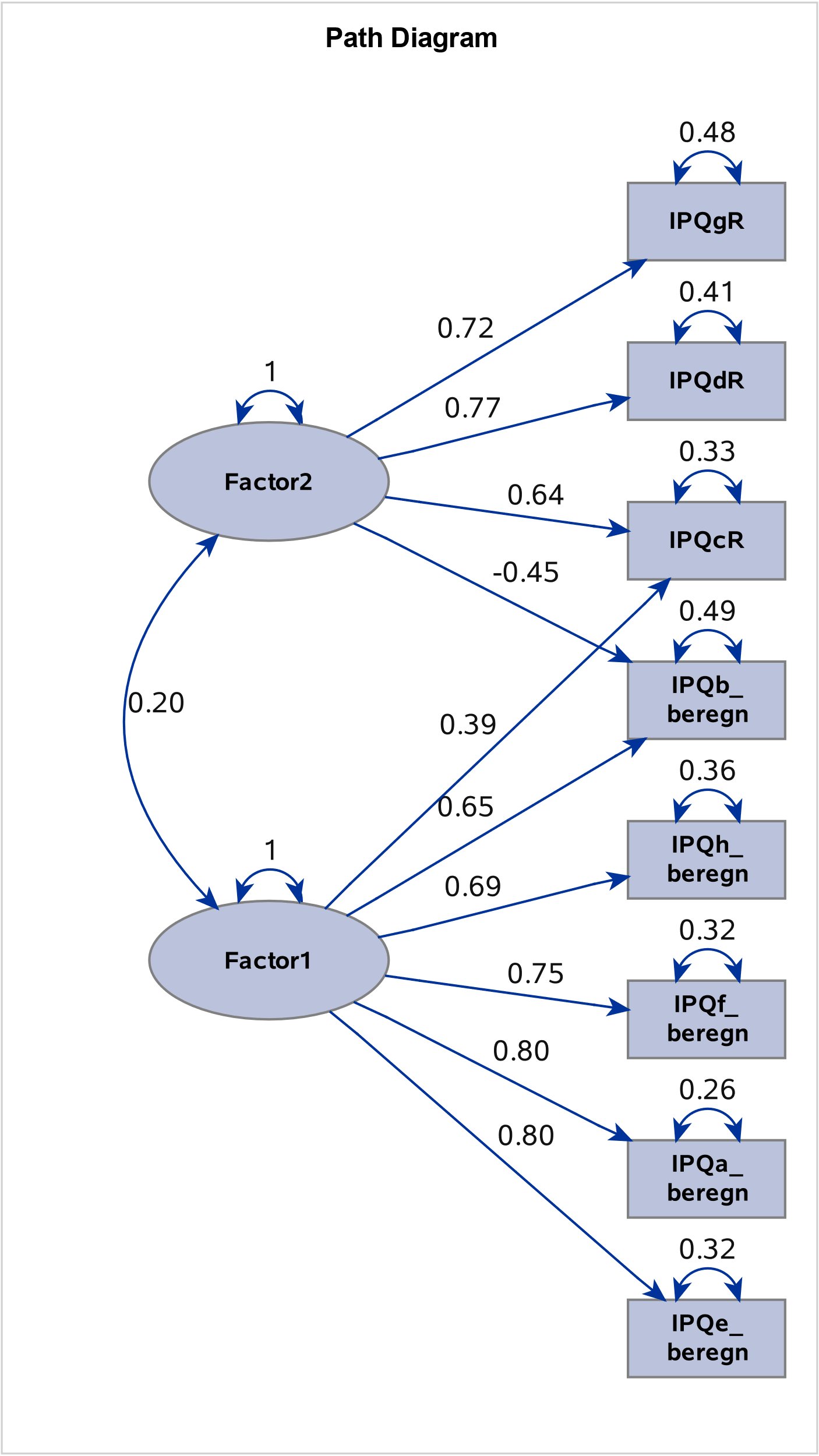


**Fig 1. Results from exploratory factor analysis: Oblique rotation**


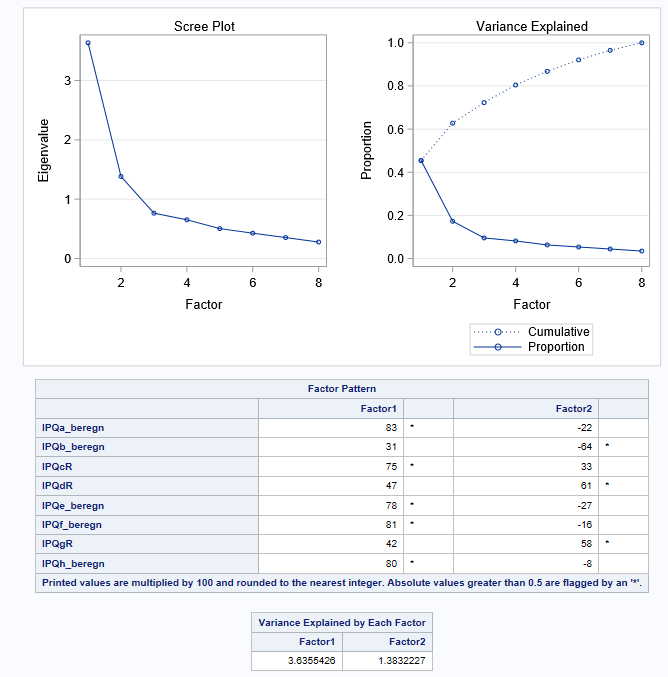


**Fig 2. Results from exploratory factor analysis: Eigenvalue Scree plot and proportion of variance explained by factors.**

**Table C. Factor analysis and domains**

| **The confirmatory factor analysis showed that a two-factor structure was acceptable. We analysed both with and without item 2 (the timeline question), and the standardised RMR, RMSEA estimate, Bentler Comparative Fit Index and Bentler-Bonett Non-Normed Index improved when this item was removed (data not shown). Therefore, we excluded item 2 and divided the IPQ scale into the following two domains:** | |
| --- | --- |
| **The Consequence domain** | **The Control domain** |
| Included question 1 (Consequences), 5 (Identity), 6 (Concern) and 8 (Emotional response) | Included question 3 (Personal control), 4 (Treatment control) and 7 (Understanding) |
| This compromises items that capture the patient’s perspective on the symptom burden (illness identity) and the impact of the disease on their health and life. It encompasses the level of concern and emotional impact experienced by the patient. Therefore, this domain reflects the patient’s thoughts and emotions regarding their current health status. | This compromises items that represent the extent to which the patient believes that they can recover from or control the illness, as well as their understanding of the disease. Thus, this domain reflects the patient’s thoughts and emotions regarding their capacity to influence their health status. |

##### **Table D1. Automated stepwise selection logistic regression with high consequence as response variable**

| **Explanatory variable** | **Score chi-square** | **P-value chi-square** |
| --- | --- | --- |
| Pain | 147.2 | < 0.01 |
| Age | 42.3 | < 0.01 |
| Physical function | 27.9 | < 0.01 |
| Available to the labour market | 10.9 | < 0.01 |
| Fatigue | 10.4 | 0.01 |
| Cohabitant status | 4.1 | 0.04 |
| Diagnosis | 5.5 | 0.07 |
| Disease activity | 5.1 | 0.08 |
| **Area under the curve** |  | **0.75** |

##### **Table D2. Automated stepwise selection logistic regression with low control as response variable**

| **Explanatory variable** | **Score chi-square** | **P-value chi-square** |
| --- | --- | --- |
| Fatigue | 76.3 | < 0.01 |
| Physical function | 30.9 | < 0.01 |
| Age | 14.9 | < 0.01 |
| Disease activity | 7.1 | 0.03 |
| Diagnosis | 5.4 | 0.07 |
| Household income | 5.4 | 0.07 |
| **Area under the curve** |  | **0.70** |
